# Change in the Treg/Th17 cell imbalance in hepatocellular carcinoma patients and its clinical value

**DOI:** 10.1097/MD.0000000000007704

**Published:** 2017-08-11

**Authors:** Yong-Ting Lan, Xiao-Peng Fan, Yu-Chen Fan, Jing Zhao, Kai Wang

**Affiliations:** a Department of Hepatology, Qilu Hospital of Shandong University, Jinan; b Department of Gastroenterology, Zibo Central Hospital, Zibo; c Institute of Hepatology, Shandong University, Jinan, China.

**Keywords:** hepatocellular carcinoma, immune mechanism, Th17, Treg

## Abstract

Recent studies have indicated that the T cell mediated immune response plays an important role in the pathogenesis of hepatitis B virus-associated hepatocellular carcinoma (HCC), but the underlying mechanism remains unclear. In this study, we found an imbalance in Treg/Th17 cells in peripheral blood mononuclear cells from HCC patients. The percentages of CD4^+^CD25^+^FOXP3^+^ Treg cells and CD4^+^IL-17^+^ Th17 cells were significantly higher in HCC patients than in the controls. The numbers of Treg and Th17 cells were increased and correlated in a positive linear manner. Moreover, the increased percentages of Treg and Th17 cells were closely related to the tumor stage and tumor size of HCC. Therefore, we concluded that Treg and Th17 cells might participate in the promotion of the invasion and progression of HCC and that a Treg/Th17 cell imbalance might be able to serve as an important indicator for determining the progression and prognosis of HCC. Further studies might provide novel therapeutic targets for HCC.

## 1 Introduction

Hepatocellular carcinoma (HCC) is a common cancer worldwide. Despite significant developments in molecular biology and cancer therapy, the overall survival of HCC patients remains low and is primarily due to distant metastasis, local recurrence, treatment resistance, and the lack of early diagnosis.^[1,2]^ HCC is closely related to hepatitis viruses, and many studies have shown that during the progression from hepatitis B virus infection to HCC, the interaction between the cellular immune response and the virus is more important than the virus itself. Therefore, it is important to explore the pathogenesis of cellular immunity in HCC. Treg and Th17 cells are derived from the same original T cells; Treg cells secrete immunosuppressive cytokines to suppress the immune response, while Th17 cells can increase the immune response via the release of inflammatory cytokines.^[3,4]^ In addition, the ratio of Treg to Th17 cells is closely related to many immune diseases, cancers, and infectious diseases.^[5]^ Many studies to date have demonstrated that the numbers of Treg and Th17 cells are increased in patients with chronic hepatitis B infection, but these changes have not been studied systematically in HCC patients. Therefore, we examined the levels of Treg and Th17 cells in the peripheral blood of HCC patients and aimed to observe the effect of a Treg/Th17 cell imbalance on the progression of HCC and to provide evidence that might aid evaluations of the prognosis and treatment guidelines of HCC.

## 2 Patients and methods

### 2.1 Study population

Serum samples were collected from 51 previously untreated HCC patients with hepatitis B virus infection from May 2013 to March 2016 at Qilu Hospital of Shandong University. Each patient's medical status was confirmed by histological diagnosis. Forty-two age- and sex-matched healthy individuals were enrolled in the control group (Table 1). Informed consent was obtained from all the patients, and the study was approved by the Institutional Review Board of Qilu Hospital of Shandong University. This study abided by the Declaration of Helsinki regarding ethical principles for medical research involving human participants.

**Table 1:**
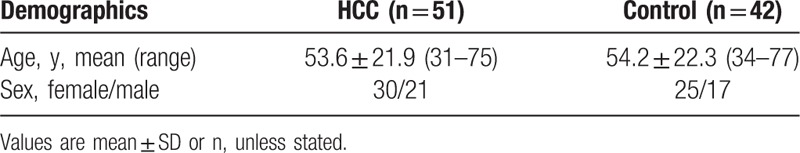
Characteristics of study populations.

The main clinical and pathological variables of the HCC patients are described in detail in Table 2. Briefly, the tumor size ranged from 1.5 to 12.0 cm (mean ± SD: 7.1 ± 3.4). According to the 7th edition of the tumor node metastasis (TNM) classification of the American Joint Committee on Cancer (AJCC),^[6]^ 10 patients had stage I disease (9 patients with a tumor size <5.0 cm and 1 with a tumor size ≥5.0 cm); 25 patients had stage II disease (21 patients with a tumor size <5.0 cm and 4 with a tumor size ≥5.0 cm); 9 patients had stage III disease (4 patients with a tumor size <5.0 cm and 5 with a tumor size ≥5.0 cm); and 7 patients had stage IV disease (1 patient with a tumor size <5.0 cm and 6 with a tumor size ≥5.0 cm).

**Table 2:**
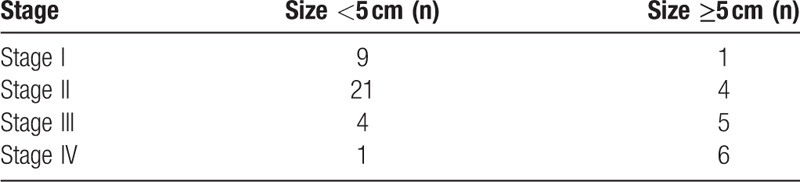
Clinicopathological information of HCC patients.

### 2.2 Assays

All the products were purchased from Affymetrix eBiosciences Company (San Diego, CA). Whole blood was collected from each patient in heparin-treated tubes at admission. For the analysis of Treg cells, PBMCs without stimulation were surface-stained with eZFluor anti-human CD4-FITC and CD25-APC Cocktail (Affymetrix, eBiosciences, San Diego, CA) and then treated with permeabilization buffer and normal rat serum, a working fixation/permeabilization solution. Intracellular staining was performed with anti-human Foxp3-PE or rat IgG2a K isotype control-PE (Affymetrix, eBiosciences, San Diego, CA) according to the manufacturer's instructions. For the analysis of Th17 cells, PBMCs were stimulated with Cell Stimulation Cocktail in complete culture medium (RPMI-1640 supplemented with 10% FBS, 200 mM L-Gln) for 5 hours at 37°C in 5% CO_2_. The cells were then incubated with anti-human CD4-FITC or mouse IgG1K isotype control-FITC (Affymetrix, eBiosciences, San Diego, CA) and then with a working fixation/permeabilization solution and permeabilization buffer. Intracellular staining was performed with anti-human IL-17A-PE or mouse IgG1K isotype control-PE (Affymetrix, eBiosciences, San Diego, CA) according to the manufacturer's instructions. FCM was performed on all the specimens within 3 hours. The data were collected on a FACSCalibur flow cytometer using CellQuest software (BD Biosciences, Franklin Lakes, NJ).

### 2.3 Statistical analysis

According to GraphPad Statmate 2.0 (GraphPad Corp, San Diego, CA), the minimum sample size in the present study was 40 samples (α = 0.05, β = 0.10). Statistical analyses were performed with GraphPad Prism 5 software (2007 GraphPad Software Inc., La Jolla, CA). The data are expressed as the means ± SD. The independent samples *t* test was used for statistical comparisons between 2 groups. A Spearman correlation analysis was used to evaluate relationships between Treg and Th17 cells, and *P* < .05 was considered statistically significant.

## 3 Results

### 3.1 Increased Treg and Th17 cells and the Treg/Th17 ratio in PBMCs of HCC patients

**Figure 1:**
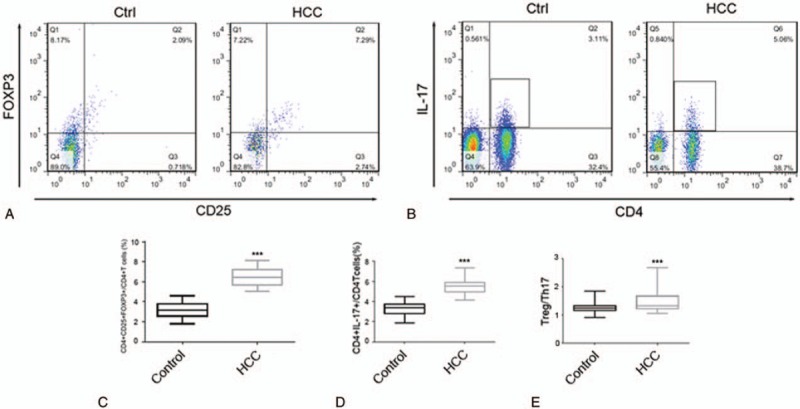
Treg and Th17 levels and the Treg/Thl7 ratio in the control group (Control) and HCC group (HCC). (A) Flow cytometric analysis of the CD4^+^CD25^+^FOXP3^+^ Treg cell levels in the control and HCC groups. (B) Flow cytometric analysis of the CD4^+^IL-17^+^ Th17 cell levels in the control and HCC groups. (C) The proportion of CD4^+^CD25^+^FOXP3^+^ Treg cells out of all CD4^+^ T cells in the control and HCC groups. (D) The proportion of CD4^+^IL-17^+^ Th17 cells out of all CD4^+^ T cells in the control and HCC groups. (E) The Treg/Th17 ratio in the control and HCC groups (^∗^*P* < .05,^†^*P* < .01,^‡^*P* < .001).

As shown in Fig. 1A and C, we found that the proportion of CD4^+^CD25^+^FOXP3^+^ Treg cells was significantly higher in the HCC group than in the control group (*P* < .001). The data in Fig. 1B and D indicated that the proportion of CD4^+^IL-17^+^ Th17 cells was markedly higher in the HCC group than in the control group (*P* < .001). As shown in Fig. 1E, the Treg/Th17 ratio was also markedly higher in the HCC group than in the control group (*P* < .001). Thus, a Treg/Th17 cell imbalance is present in HCC patients and is involved in the pathogenesis of HCC.

### 3.2 Increased numbers of Treg and Th17 cells in PBMCs of advanced-stage HCC patients

**Figure 2:**
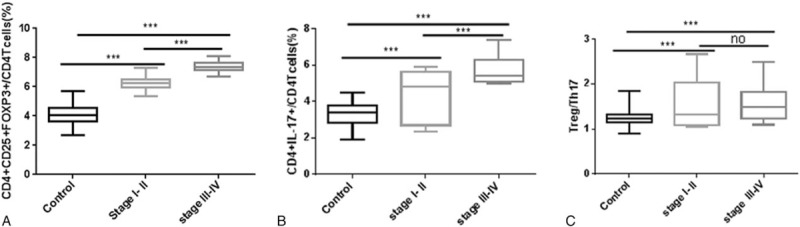
Treg and Th17 levels and the Treg/Thl7 ratio in the control group (Control), early-stage HCC group (stage I-II), and advanced-stage HCC group (stage III-IV). (A) The proportion of CD4^+^CD25^+^FOXP3^+^ Treg cells out of all CD4^+^ T cells in the control, stage I-II, and stage III-IV groups. (B) The proportion of CD4^+^IL-17^+^ Th17 cells out of all CD4^+^ T cells in the control, stage I-II, and stage III-IV groups. (C) The Treg/Th17 ratio in the control, stage I-II, and stage III-IV groups (“no” refers to *P* > .05,^∗^*P* < .05,^†^*P* < .01,^‡^*P* < .001).

As shown in Fig. 2A, the proportion of CD4^+^CD25^+^FOXP3^+^ Treg cells was significantly higher in the different HCC tumor stage groups than in the control group; moreover, the proportion of CD4^+^CD25^+^FOXP3^+^ Treg cells was significantly higher in the advanced (stage III-IV) HCC group than in the early (stage I–II) HCC group. As indicated in Fig. 2B, the proportion of CD4^+^IL-17^+^ Th17 cells was significantly higher in the different HCC tumor stage groups than in the control group; moreover, the proportion of CD4^+^IL-17^+^ Th17 cells was significantly higher in the advanced (stage III-IV) HCC group than in the early (stage I-II) HCC group. As shown in Fig. 2C, the Treg/Th17 ratio was significantly higher in the different HCC tumor stage groups than in the control group; however, no difference was observed in the Treg/Th17 ratio in the advanced (stage III-IV) HCC group or in the early (stage I-II) HCC group.

### 3.3 Associations between the frequencies of Treg and Th17 cells and the clinical characteristics of HCC patients

**Figure 3:**
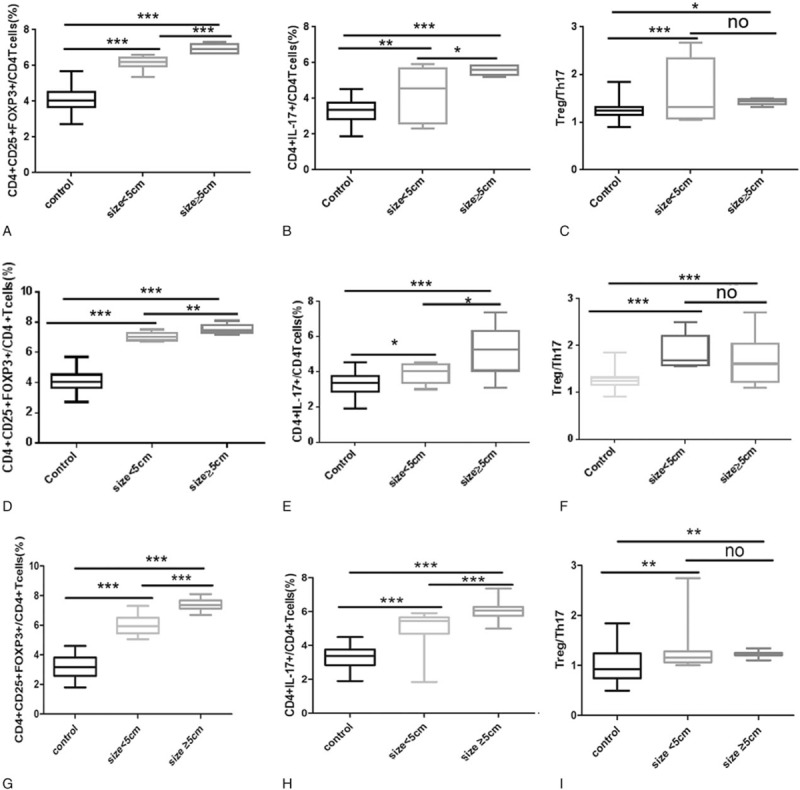
Treg and Th17 levels and the Treg/Th17 ratio in the control group (Control), the small tumor size HCC group (size < 5 cm), and the large tumor size HCC group (size ≥5 cm). (A) The proportion of CD4^+^CD25^+^FOXP3^+^ Treg cells out of all CD4+ T cells in the control, size < 5 cm (early stage I-II) and size ≥5 cm (early-stage I-II) groups. (B) The proportion of CD4^+^IL-17^+^ Th17 cells out of all CD4^+^ T cells in the control, size <5 cm (early-stage I-II) and size ≥5 cm (early-stage I-II) groups. (C) The Treg/Th17 ratio in the control, size <5 cm (early stage I-II) and size ≥5 cm (early-stage I-II) groups. (D) The proportion of CD4^+^CD25^+^FOXP3^+^ Treg cells out of all CD4+ T cells in the control, size <5 cm (advanced-stage III-IV) and size ≥5 cm (advanced-stage III-IV) groups. (E) The proportion of CD4^+^IL-17^+^ Th17 cells out of all CD4^+^ T cells in the control, size <5 cm (advanced-stage III-IV) and size≥5 cm (advanced-stage III-IV) groups. (F) The Treg/Th17 ratio in the control, size<5 cm (advanced-stage III-IV) and size≥5 cm (advanced-stage III-IV) groups. (G) The proportion of CD4^+^CD25^+^FOXP3^+^ Treg cells out of all CD4+ T cells in the control, size<5 cm and size≥5 cm groups. (H) The proportion of CD4^+^IL-17^+^ Th17 cells out of all CD4^+^ T cells in the control, size<5 cm and size≥5 cm groups. (I) The Treg/Th17 ratio in the control, size<5 cm and size≥5 cm groups (“no” refers to *P* > .05,^∗^*P* < .05,^†^*P* < .01,^‡^*P* < .001).

As shown in Fig. 3A, the proportion of CD4^+^CD25^+^FOXP3^+^ Treg cells was significantly higher in the early-stage (I–II) HCC groups of patients with various tumor sizes than in the control group; moreover, the proportion of CD4^+^CD25^+^FOXP3^+^ Treg cells was significantly higher in the HCC group with large tumors than in the HCC group with small tumors. The data in Fig. 3B indicated that the proportion of CD4^+^IL-17^+^ Th17 cells was significantly higher in the HCC groups of early-stage (I–II) patients with various tumor sizes than in the control group; moreover, the proportion of CD4^+^IL-17^+^ Th17 cells was significantly higher in the HCC group of patients with large tumors than in the HCC group of patients with small tumors. As shown in Fig. 3C, the Treg/Th17 ratio was significantly higher in the HCC groups of early-stage (I–II) patients with different tumor sizes than in the control group; however, no difference was observed in the Treg/Th17 ratio between the HCC group with large tumors and the HCC group with small tumors.

As shown in Fig. 3D, we found that the proportion of CD4^+^CD25^+^FOXP3^+^ Treg cells was significantly higher in the advanced-stage (III–IV) HCC groups of various tumor sizes than in the control group; moreover, the proportion of CD4^+^CD25^+^FOXP3^+^ Treg cells was significantly higher in the large tumor size HCC group than in the small tumor size HCC group. As shown in Fig. 3E, the proportion of CD4^+^IL-17^+^ Th17 cells was significantly higher in the advanced-stage (III–IV) HCC groups of various tumor sizes than in the control group; moreover, the proportion of CD4^+^IL-17^+^ Th17 cells was significantly higher in the large tumor size HCC group than in the small tumor size HCC group. The data in Fig. 3F indicated that the Treg/Th17 ratio was significantly higher in the advanced-stage (III-IV) HCC groups of different tumor sizes than in the control group; however, no difference was observed in the Treg/Th17 ratio between the large- and small tumor size HCC groups.

As shown in Fig. 3G, the proportion of CD4^+^CD25^+^FOXP3^+^ Treg cells was significantly higher in the HCC groups of patients with various tumor sizes than in the control group; moreover, the proportion of CD4^+^CD25^+^FOXP3^+^ Treg cells was significantly higher in the HCC group with a large tumor size than in the HCC group with a small tumor size. The data in Fig. 3H indicated that the proportion of CD4^+^IL-17^+^ Th17 cells was significantly higher in the HCC groups of various tumor sizes than in the control group; moreover, the proportion of CD4^+^IL-17^+^ Th17 cells was significantly higher in the HCC group with a large tumor size than in the HCC group with a small tumor size. As shown in Fig. 3I, the Treg/Th17 ratio was significantly higher in the HCC groups of different tumor sizes than in the control group; however, no difference was observed in the Treg/Th17 ratio between the HCC group with a large tumor size and the HCC group with a small tumor size.

As summarized in Table 3, no significant difference was found in the proportion of CD4^+^CD25^+^FOXP3^+^ Treg cells between male and female HCC patients (*P* > .05). In addition, no significant difference (*P* > .05) was observed in the proportion of CD4^+^CD25^+^FOXP3^+^ Treg cells between older (≥60 years of age) and younger HCC patients (<60 years of age). As indicated in Table 4, no significant difference was found in the proportion of CD4^+^IL-17^+^ Th17 cells between male and female HCC patients (*P* > .05). Moreover, no significant difference (*P* > .05) was observed in the proportion of CD4^+^IL-17^+^ Th17 cells between older (≥60 years of age) and younger HCC patients (<60 years of age).

**Table 3:**
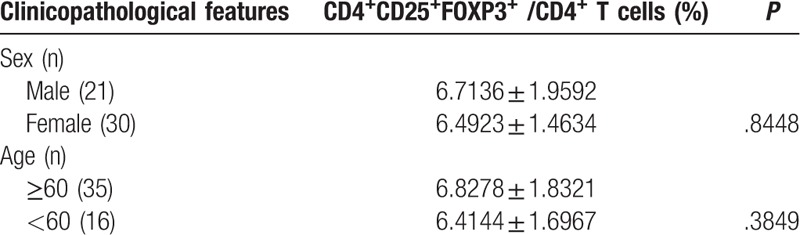
The correlations between the Treg cells and the patient characteristics.

**Table 4:**
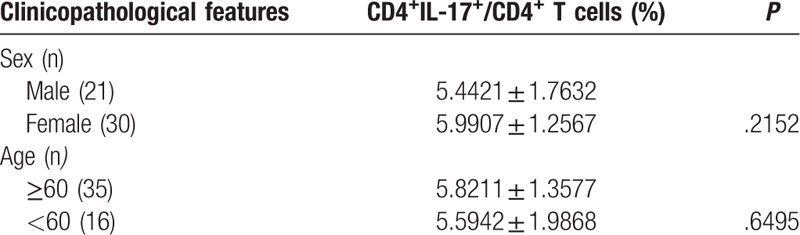
The correlations between the Th17 cells and the patient characteristics.

### 3.4 Positive linear correlation between Treg and Th17 cells in the PBMCs of HCC patients

**Figure 4:**
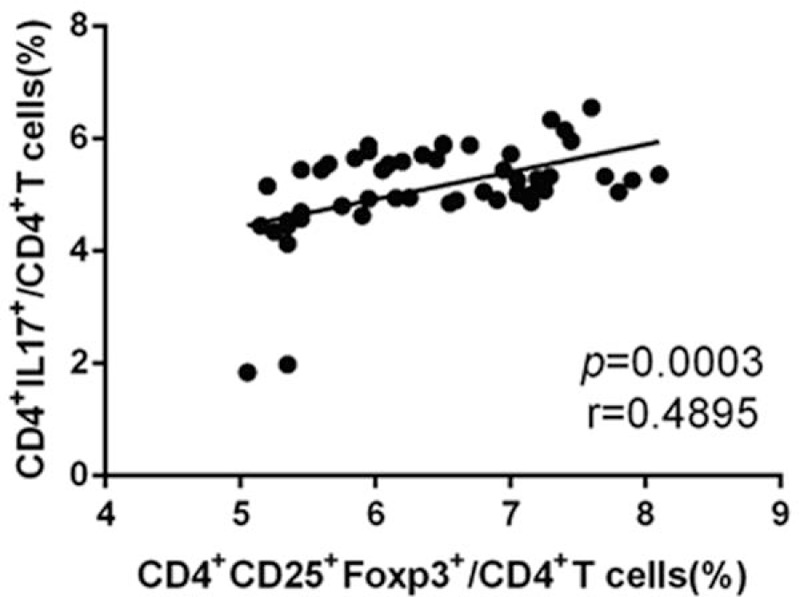
The levels of Treg and Th17 cells in the peripheral blood of HCC patients. The levels were increased in a positive linear manner, *r* = 0.4895, *P* < .001, n = 51.

Because the proportions of CD4^+^CD25^+^FOXP3^+^ Treg cells and CD4^+^IL-17^+^ Th17 cells were markedly higher in the stage III-IV group than in the stage I-II group, we used Spearman correlation analysis to study the correlation between Treg and Th17 cells in the peripheral blood of patients in the HCC group. A positive linear correlation was observed between Treg and Th17 cells (Fig. 4; *r* = 0.4895, *P* < .001, n = 51). This result indicated that the Treg/Th17 ratio was associated with the invasion and progression of HCC and might serve as a marker for the diagnosis of HCC, that is, a higher Treg/Th17 ratio is associated with a worse prognosis for HCC patients.

## 4 Discussion

The main cause of HCC in China is hepatitis B virus infection. The immune response is considered an important mechanism for the occurrence and progression of HCC. An increasing number of studies has shown that the immune response mediated by T lymphocytes plays an important role in anti-tumor immunity.^[7]^ Both Treg and Th17 cells are important subtypes of CD4^+^ T lymphocytes. Although Treg cells play a fundamental role in immunosuppression, Th17 cells play the opposite role. A Treg/Th17 imbalance might cause reduced immunity, immune tolerance, or even the immune escape of tumor cells.

CD4^+^CD25^+^ Treg cells contribute to immunological hyporesponsiveness and immune suppression,^[8]^ and therefore, these cells are involved in the maintenance of immune homeostasis and prevent the occurrence of autoimmune diseases. Moreover, CD4^+^CD25^+^ Treg cells play an important role in the regulation of tumor immunity.^[9,10]^ Ormandy et al^[11]^ reported that the level of Treg cells in the peripheral blood of HCC patients was significantly higher than that in the peripheral blood of healthy individuals. In the present study, we obtained the same result. We found that the peripheral blood levels of CD4^+^CD25^+^FOXP3^+^ Treg cells in HCC patients were significantly higher than those of the control group (*P* < .001), which indicates that the immune response mediated by CD4^+^CD25^+^ Treg cells was closely related to the pathogenesis of HCC. Moreover, it was found that the levels of CD4^+^CD25^+^FOXP3^+^ Treg cells in the peripheral blood of advanced (stage III-IV) HCC patients were higher than those of early (stage I-II) HCC patients, which implies that the presence of CD4^+^CD25^+^ Treg cells was closely related to tumor progression. Further studies revealed that the level of CD4^+^CD25^+^ Treg cells was closely related to tumor size. In early-stage (stage I-II) HCC patients, the levels of CD4^+^CD25^+^FOXP3^+^ Treg cells in the peripheral blood of patients with large tumors were significantly higher than those of patients with small tumors. In advanced-stage (stage III-IV) HCC patients, the levels of CD4^+^CD25^+^FOXP3^+^ Treg cells in the peripheral blood of those with large tumors were significantly higher than those with small tumors. In the HCC group, the levels of CD4^+^CD25^+^FOXP3^+^ Treg cells in the peripheral blood of patients with large tumors were significantly higher than those with small tumors. This study revealed that, with the progression of HCC, the proportion of CD4^+^CD25^+^ Treg cells was increased. We may conclude that Treg cells exerted a promoting effect on the invasiveness and metastasis of HCC. Therefore, the number of Treg cells in the peripheral blood may be a potential marker for the diagnosis and prognosis of HCC patients.

In most cases, CD4^+^CD25^+^ Treg cells suppress the anti-tumor immune response in 2 aspects: one mode is via cells in the tumor-draining regional lymph node; the other mode is through the tumor tissue. In the tumor-draining regional lymph node cells, many proliferative CD4^+^CD25^+^ Treg cells inhibit the proliferation of effector cells within the same lymph node. In the tumor tissue, CD4^+^CD25^+^ Treg cells prevent effector T cells from killing tumor cells.^[12]^ Zhou et al^[13]^ found that the level of Treg cells in cancer tissue was significantly higher than that in adjacent tissues. In the tumor microenvironment (TME), Treg cells might decrease the proliferation of CD4^+^ T lymphocytes by cell contact inhibition, which would reduce the local immune response to tumors such that the tumor cells could escape immune surveillance. In conclusion, the removal or reduction of the Treg cell population in the HCC microenvironment may facilitate the effect of tumor immunotherapy.

Th17 cells are a recently discovered subtype of helper T cells. This subtype is involved in the pathological process of tumors, host defence, infection, autoimmune diseases, and transplant rejection through the secretion of certain cytokines, such as interleukin (IL)-17A, IL-17F, IL-21, IL-22, and IL-6. IL-17A is the most important cytokine, as the receptor of IL-17A is widely expressed in vivo. However, the function of Th17 cells in tumor pathogenesis remains unclear. On the one hand, Th17 cells can induce the production of IL-6; this activates the oncogene signal transducer and activator of transcription (STAT) 3, which then upregulates genes that induce tumor angiogenesis. Th17 cells can thus promote tumor growth and metastasis.^[14]^ On the other hand, through the secretion of IL-17 and interferon-gamma (IFN-γ), which increase the expression of cytotoxic lymphocytes (CTLs) in the TME, Th17 can inhibit tumor growth.^[15]^ Zhang et al^[16]^ found that the level of Th17 cells in tumor tissues was significantly higher than that in non-neoplastic tissues and that the density of Th17 cells in tumor tissue had a negative linear correlation with the overall survival of patients with HCC. In this study, we found that the number of CD4^+^IL-17^+^ Th17 cells was significantly higher in the peripheral blood of patients in the HCC group than that of patients in the control group. Moreover, the presence of Th17 cells was closely related to tumor stage. The level of CD4^+^IL-17^+^ Th17 cells in advanced-stage (III-IV) HCC patients was higher than that of those with early-stage (I-II) disease. Further studies revealed that the presence of Th17 cells was closely related to tumor size. In the early-stage (I-II) group and the advanced-stage (III-IV) group, the levels of CD4^+^IL-17^+^ Th17 cells in the peripheral blood of patients with large tumors were significantly higher than those in the blood of patients with small tumors. In the HCC group, the levels of CD4^+^IL-17^+^ Th17 cells in the peripheral blood of patients with large tumors were significantly higher than those in the blood of patients with small tumors. This result indicated that Th17 cells may exert negative immunomodulatory effects on local immunity in HCC and that Th17 cells may be a promising target for HCC treatment.

In addition, many studies have shown that the incidence and prognosis of HCC is closely related to patient characteristics, such as age and sex, among other characteristics. The aging of the population is closely associated with the increased mortality of HCC patients.^[17]^ Compared with male HCC patients, most female HCC patients have the following features: complete tumor capsule, high survival rate, low recurrence, and a good prognosis.^[18]^ Therefore, in this study, we analyzed the correlations between Treg and Th17 cells with age and sex. However, no significant statistical results were found (*P* > .05). Due to the limitations in the sample size in this study, the relationship between Treg and Th17 cells with patient characteristics in this field needs to be explored further.

Th17 cells and Treg cells restrain each other in terms of their function. In autoimmune diseases, immune populations of Treg/Th17 are imbalanced, that is, if one is upregulated, the other is downregulated. In our study, we found that both Treg and Th17 cell numbers increased with the progression of HCC; further investigation found a positive linear correlation between Treg and Th17 cells. The change in the Treg/Th17 ratio may be attributed to the specific TME of HCC. The TME is the cellular environment in which the tumor exists and includes the surrounding blood vessels, extracellular matrix (ECM), and tumor stromal cells,^[19]^ such as T cells, myeloid-derived suppressor cells (MDSCs), macrophages, and mast cells. As an important type of innate immune cell in the TME, mast cells can increase the expression of CCL_2_ and thereby attract massive numbers of MDSCs.^[20]^ MDSCs can produce IL-17, which recruits Treg cells via an increase in the chemotactic factors CCL_17_ and CCL_22_. In addition, a large number of endogenous Toll-like receptor (TLR) agonists may be found within the TME, such as the degradation products of HMGB1 (High Mobility Group Box 1) and products of lipid metabolism.^[21]^ TLR is a type I transmembrane protein. As one type of pattern recognition receptor (PRR), TLRs can identify the conservative components of invasive pathogenic microorganisms.^[22]^ TLRs are widely expressed on Treg and Th17 cells. Endogenous TLR agonists can promote immune escape through the activation of Treg cells.^[23,24]^ In addition, these agonists may act on Th17 cells directly and then promote the transformation of Th17 cells.

In conclusion, the levels of Treg and Th17 cells and the Treg/Th17 ratio were higher in the peripheral blood of HCC patients than in the peripheral blood of controls. Treg and Th17 cells were closely related to the tumor stage and tumor size, and a positive linear correlation was observed between Treg and Th17 cells. These results showed that an imbalance in Treg/Th17 cells was closely related to the progression and prognosis of HCC. The Treg/Th17 ratio could be a promising candidate for the diagnosis and prognosis of HCC patients. Therefore, considering the close relationship among Treg cells, Th17 cells, and HCC, the removal or reduction of Treg and Th17 cells from the TME might increase the effect of tumor immunotherapy and improve the final outcome of HCC patients. Further large-scale, multicenter studies are needed to demonstrate additional associations between Treg and Th17 cells and HCC.
